# Study on the potential capacity of cake fertilizer agricultural solid emission reduction and soil improvement based on CiteSpace

**DOI:** 10.3389/fpls.2025.1400159

**Published:** 2025-04-03

**Authors:** Yuliang Fu, Gang Li, Songlin Wang, Zhiguang Dai, Xiaoyuan Zhang

**Affiliations:** ^1^ School of Water Conservancy, North China University of Water Resources and Electric Power, Zhengzhou, China; ^2^ College of Agricultural Equipment Engineering, Henan University of Science and Technology, Luoyang, China

**Keywords:** cake fertilizers, CiteSpace, soil improvement, greenhouse gas, potential capacity

## Abstract

**Introduction:**

The goal was to gain a comprehensive understanding of the current research status, hotspots and cutting-edge dynamics in the field of cake fertilizer application in agriculture at home and abroad from 2000 to 2024.

**Methods:**

The study employed the bibliometric analysis method and CiteSpace visualisation software to investigate the research results of the field of cake fertilizer agriculture in greenhouse gases and soil improvement included in the Core Collection Database of Web of Science.

**Results:**

The findings of the keyword analysis, collaborative network analysis, and publication count analysis demonstrated that (1) "The number of related literatures was small and in its infancy before 2007,accounting for 17% of the total number of publications; the slow growth phase was from 2008-2016, accounting for 39% of the total number of publications(with a growth rate of 1.65%); and the rapid increase phase was after 2017-2023, accounting for 39% of the total number of publications(with a growth rate of 3.89%). (2) India ranked first in terms of the number of publications, and China ranked second in terms of the number of publications, but China was first in terms of intermediary centrality, and the authors were all loosely distributed, choosing to publish their research results in international journals with an impact factor of greater than 2 in the field of agriculture. (3) Universities are the primary source of research findings in this field among the issuing institutions. (4) The research hotspots include nitrogen, soil, growth, yield, manure, fertilizer, quality, management; The research themes with the greatest number of keywords are "bag filiter," and "bacillus sp " has the highest profile value; The development trends are as follows: prior to 2016, the fertilizer program studied various organic fertilizers in combination with chemical fertilizers or different types of organic fertilizers to explore the impact on crop yields; subsequent to 2016, the fertilizer program studied cake fertilizers with new materials to evaluate the impact on crop yield quality and soil fertility. Going forward, the emphasis should be on blending environmentally friendly components with cake fertilizers and carrying out comprehensive studies on soil enhancement and greenhouse gas mitigation strategies.

**Discussion:**

This study offers new insights and ideas on the future research direction and development potential of cake fertilizer in agricultural greenhouse gas emission reduction and soil improvement. It also serves as a valuable reference for understanding the development trend of cake fertilizer application in agriculture from 2000 to 2023.

## 1 Introduction

Climate change is one of the biggest problems the world is now experiencing and it severely threatens human life and productivity (Wu et al., 2023; Zhao et al., 2023). Energy use, industry, transport and agriculture are essential drivers of climate change (Leitão and Balogh, 2020). Agriculture is a pivotal contributor to greenhouse gas emissions, accounting for approximately one-third of all such emissions (Fuentes Ponce et al., 2022). In agricultural production, greenhouse gases are generated directly during the sowing and growing of crops (Fan et al., 2022), and agriculture requires direct energy inputs in the form of fuels to power agricultural machinery, and equipment and to heat or cool buildings. In addition, the production of agricultural fertilizers and other chemicals contributes indirectly to greenhouse gas emissions (Wang et al., 2017; Ghimire et al., 2024). This is due to the fact that N_2_o is created in part by soil nitrification and in part as an intermediate product of soil denitrification during the breakdown and absorption of nitrogenous fertilizers by the soil (He and Loffler, 2024). It is therefore important that we increase our efforts to reduce the use of chemical fertilizers and promote green and high-quality agriculture to mitigate the effects of climate change and ensure sustainable food production in the future (Lin TY. et al., 2022). As global demand for agricultural products is anticipated to quadruple by 2050, owing primarily to rising living standards, we must address the environmental implications of our food production and consumption (Rojas-Downing et al., 2017).

Research indicates that the use of organic fertilizers can improve the ammonification process and harmonize the nitrification and denitrification processes, which in turn lowers the production of N_2_o and ammonia volatilization (Chen et al., 2020). Thus, there is a significant potential that using organic fertilizers will lower greenhouse gas emissions (Muller et al., 2017). Increased crop yields are another obvious benefit of using organic fertilizers, and this benefit is mostly derived from better soil conditions (Wang et al., 2019; Ye et al., 2020). Organic fertilizer has two main effects: on the one hand, organic fertilizer can supply crops with the essential nutrient sources, boost soil nutrients (Fu et al., 2024), and enhance soil structure (Liu et al., 2019). The structure and function of the soil microbial community can be optimized by using organic fertilizers (Sun et al., 2023), on the other hand, which can also encourage the growth and reproduction of microorganisms (Jiang et al., 2022). This increases the soil’s metabolic capacity and nutrient conversion efficiency, making it easier for crops to absorb and utilize soil nutrients (Li et al., 2020). It is noteworthy that cake fertilizer, as a type of organic fertilizer, has also received attention from researchers (Mirpoor et al., 2021).

Cake fertilizer, which is high in nitrogen, carbohydrates, and protein, is the byproduct of oil extraction from oilseed crop seeds (de Almeida Junior et al., 2011). Globally, the main oilseed crops are soybean, rapeseed, peanut and sunflower are the main oilseed trade varieties (Alzamel et al., 2022). Therefore, soybean cake fertilizer, rapeseed cake fertilizer, peanut cake fertilizer and sunflower cake fertilizer are used all over the world. Noteworthily, India, being the largest producer of oilseeds globally, manufactures more than 25 million metric tons of oilseed cakes every year (Singh et al., 2022). In the global market, soybean cake stands out as the most prominent oilcake/meal product, accounting for 54% of total production. Rapeseed cake/meal follows closely, making up 10% of the total output (Vichare and Morya, 2024). It has been discovered that cake fertilizers are primarily utilized as supplements for animal feed and soil (Mirpoor et al., 2021), and they are also used to medications (Zhao et al., 2017), films (Aramwit et al., 2022), and lubricants (Sahin et al., 2017). When applied directly to animals as feed, cake fertilizer has reportedly been shown to be poisonous (Worbs et al., 2011). Still, it can only be used as feed after detoxification of the cake fertilizer by chemical, physical, biological, or combined processes (Gomes et al., 2018). Currently, cake fertilizers are not only used as feed for poultry but are also fully utilized in fisheries (Mredul et al., 2022; He et al., 2024). However, cake fertilizer can be used directly as a soil fertilizer after fermentation to provide nutrients to the soil (Song et al., 2023). It is commonly recognized that the use of cake fertilizer in medications, flicks, additives, etc. necessitates the extraction of compounds unique to the fertilizer, which is an expensive and difficult procedure to carry out (Jingura and Kamusoko, 2018). However, cake fertilizer can be used directly as a soil fertilizer to provide nutrients to the soil (Jung and Choi, 2020). In addition, different cake fertilizers have varying chemical compositions, qualities, growth conditions, extraction techniques, and storage parameters (Martin et al., 2010). They also exhibit significant instability in the process of extracting the necessary substances. Furthermore, it is sufficient to apply fertilizer in accordance with the kind of cake fertilizer manufactured locally; it is not essential to take the type of cake fertilizer into consideration. This is due to various cake fertilizers can all increase soil fertility, they can only do so by enhancing the various nutrients already present in the soil (Xu et al., 2020).Therefore, when compared with the application of cake fertilizer in other aspects, the application of cake fertilizer as soil fertilizer has a significant advantage.

Cake fertilizer has the highest soil enzyme activity of any organic fertilizer when compared to amino acid eco-fertilizer and chicken type fertilizer treatments. Soil dormant enzyme activity, soil acid phosphatase, soil sucrase and soil dehydrogenase were all significantly increased after 150 days of cake fertilizer treatment (Li et al., 2014). AdeOluwa et al. studied the effects of Jatropha seed cake (JSC), Tithonia (Tithonia diversifolia), compost, and the other most common compound inorganic fertilizers in Nigeria-NPK on cucumber (AdeOluwa et al., 2021). This proved that the cake fertilizer was significantly increased. According to Wang et al., the nine-year average rice yield of cake fertilizer treatment was 60.0% higher than no fertilizer, and the total nitrogen content was 0.23-0.85 g kg^-1^ higher than other treatments, respectively, based on a field experiment (Wang et al., 2023). Shan et al. demonstrated that the application of canola cake fertilizer together with straw mulching treatment had a significant effect on the soil fertility of the tea plantation. Soil organic matter content, total nitrogen content, effective phosphorus content, and the number of microorganisms, such as aminobacteria, aerobic autochthonous nitrogen-fixing bacteria, and smoky autochthonous nitrogen-fixing bacteria were significantly higher than that produced during the treatment of purely applying chemical fertilizer (Shan et al., 2010). Cake fertilizer provides an appropriate organic matter and fungal development environment in the soil, efficiently enhances its soil ecological balance, and serves as an excellent material basis for additional cake fertilizer for soil improvement.

In summary, the benefits of returning cake fertilizer to the field include: 1. increasing soil enzyme activity and nutrient content; 2. increasing crop yield and quality; 3. optimizing the soil microbial community. The drawbacks of returning cake fertilizer to the field include: 1. the need for chemical or biological detoxification of some cake fertilizers due to their natural toxins; 2. the wide variation in nutrient ratios and toxin contents among different cake fertilizers, which affects the consistency of the fertilizer application effect.

At this point, some academics are interested in the impact of cake fertilizer on agricultural greenhouse gas emissions. For example, Zhang et al. conducted a comparative analysis of the effects of different types of organic fertilizers and chemical fertilizers on greenhouse gas emissions, and confirmed that in the wheat-soybean replanting system, the distribution of cake fertilizer and chemical fertilizer significantly reduce the net greenhouse effect (Zhang et al., 2020). Furthermore, Meng discovered that mixing cake fertilizer could significantly reduce the cumulative emissions of soil N_2_o (Meng, 2021). Although the great potential of cake fertilizer in reducing agricultural greenhouse gas emissions has been demonstrated, key technologies such as the regulatory mechanisms and causes of inhibition of cake fertilizer have not been systematically analyzed. Therefore, enhancing research on agricultural greenhouse gases from cake fertilizer and directing the technical system for regulating these gases can aid in comprehensively grasping the regulatory mechanism for cake fertilizer in agricultural greenhouse gas emissions. This endeavor is essential for advancing the sustainable development of cake fertilizer in managing agricultural greenhouse gases.

Little has been reported on its systematic sorting and visual analysis. In view of this, this paper is based on the core database of Web of Science (WoS), and through CiteSpace, the number of articles, countries, research institutions, published journals, and keyword analysis of greenhouse gases in cake fertilizer agriculture and soil improvement are analyzed. The analysis is aimed at further exploring the research contents, status and hotspots of greenhouse gas regulation and soil improvement in cake fertilizer agriculture, tapping the potential of this research field, and pointing out the direction for future research and development.

## 2 Data sources and research methodology

### 2.1 Data sources

**Figure 1:**
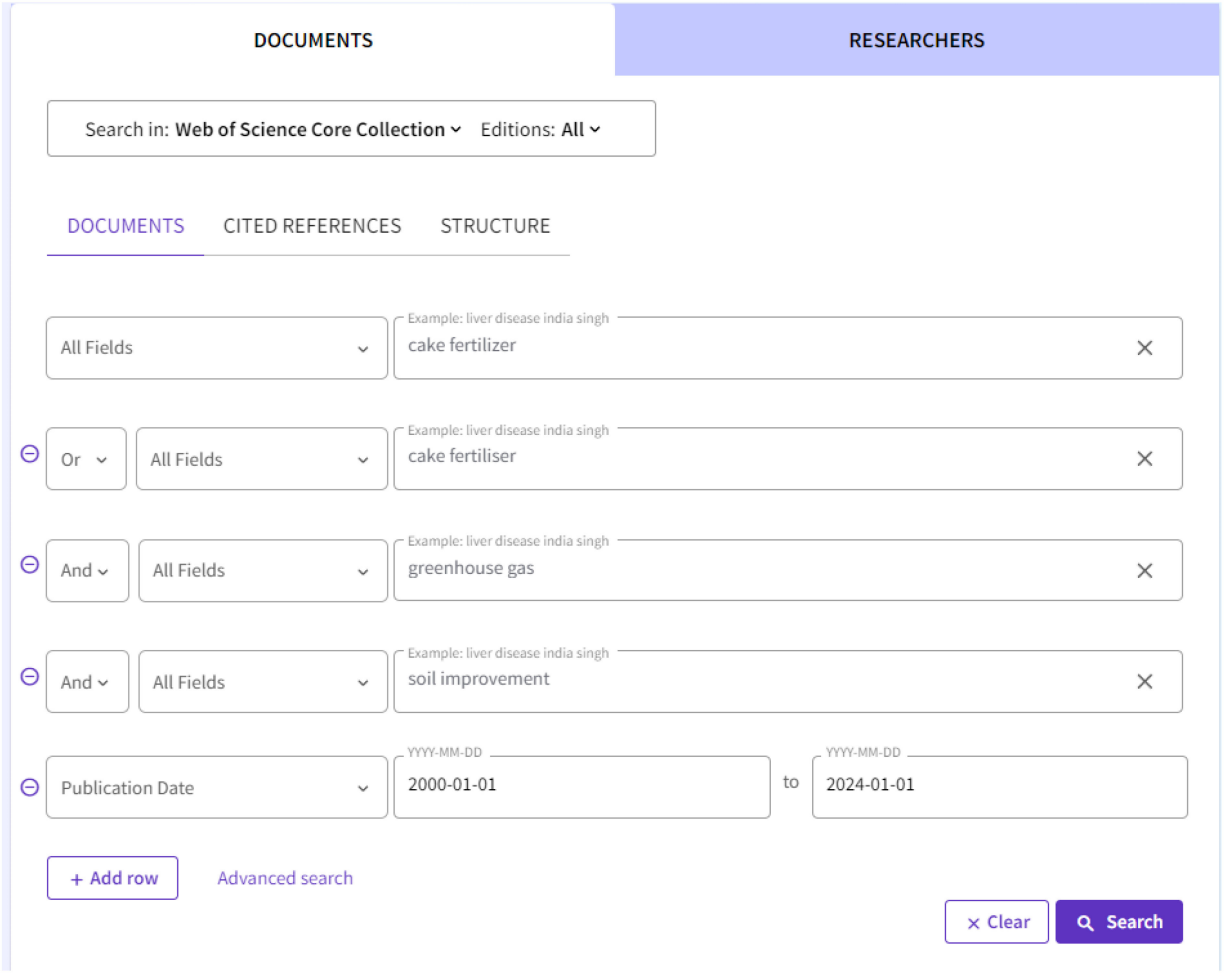
Data retrieval details.

The data in this paper were retrieved from the Web of Science Core Collection [https://www.webofscience.com]. The search for cake fertilizer, cake fertilizer, greenhouse gases and soil improvement (**Figure 1**) totaled 679 papers. Literature exclusion criteria: non-agricultural field applications essay, duplicate papers, conference papers, scientific and technical results, newspaper literature. There were a total of 481 documents. After the subsequent CiteSpace de-duplication process, the available literature remained at 481 papers. The time period was from 1 January 2000 to 1 January 2024, and the data were latest updated on 10 September 2024.

### 2.2 Research methodology

In this study, the bibliometric methodology was used to obtain the relationships between the research components through scientific mapping using CiteSpace (Wu et al., 2019). The lines of development of the research on cake fertilizer in agriculture were summarized (Tian and Chen, 2021). On the one hand, a collaborative network analysis methodology was used to illustrate the spatial representation of the interrelationships between the authors and the keywords for identifying the collaboration and interactions between the researchers who collaborate in the particular research area. Collaboration among researchers can lead to increased clarity of the subject matter, the production of richer perspectives, and the chance to form new research groups; on the other hand, co-occurrence network analysis (Yang et al., 2023) is used for detecting author keyword analyses. Co-occurrence network analysis frequently reveals thematic homogeneity with concurrent keywords, guiding future researchers to associate with groups that exhibit richer expression in the subject field. The node size of the co-occurrence network analysis represents the frequency of keywords and themes appearing, and the connecting line between the nodes indicates the strength of co-occurrence. The data were visualized and analyzed in terms of country, main author, institution, published journal, keyword analysis, and co-cited literature.

CiteSpace is a Java-based information visualization software that maps visual scientific knowledge through a series of theories to elucidate patterns of cooperation, cognitive and intellectual structures, and the definition and evolution of scientific fields (Chen, 2013; Chen and Song, 2019). CiteSpace has a powerful timeline analysis that clearly shows the evolution of research hotspots (Zhang et al., 2022). CiteSpace can precisely observe the articles that are part of a certain node, the size and content of the clusters, and the average year of the clusters in the results that are displayed (Li et al., 2022). Consequently, the scientific quality, precision, and clarity of CiteSpace’s study of the scientific knowledge graph are noteworthy.

CiteSpace parameter settings (Wang et al., 2022): 1. January 2000 to January 2024 is the time frame for the analysis of Chinese and English literature. 2. The node type is chosen according on the content being analyzed, and it is chosen just once.3. In this paper, the value of the K is set to 25, which is a scale factor, and the size of the network is modified by raising or reducing the scale parameter the K. The greater the K, the larger the network. 4. the threshold positioning is Top 50. 5. In Pruning, select Pruning to merge networks.

This paper uses version CiteSpace 6.2. R6Advanced with Wps Office 2019 for mapping and analysis.

## 3 Results and analyses

### 3.1 Temporal changes in the volume of published research literature

**Figure 2:**
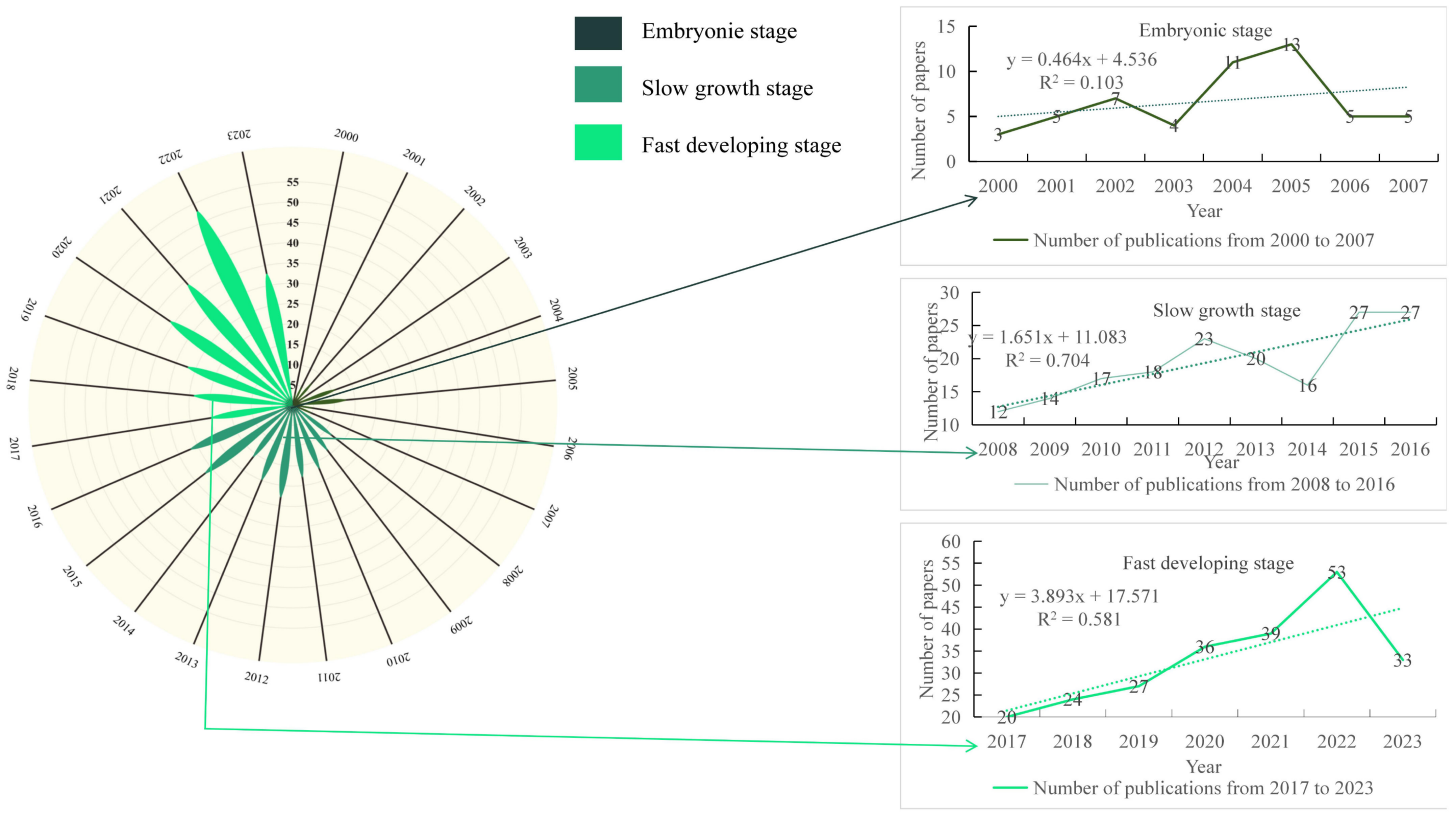
Number of publications on solid-state emission reduction and soil improvement of cake fertilizer agriculture from 2000 to 2024.

Based on the year of publication of the literature, the analysis obtained the change of the number of publications on greenhouse gases and soil improvement in cake fertilizer agriculture from 2000 to 2024 as shown in **Figure 2**. The number of related publications was low before 2007, after which the number of publications showed a general growth trend. With 2016 as the watershed, 2007-2016 was a slow growth stage (with a growth rate of 1.65%), accounting for 39% of the total number of publications. And after 2017-2022 was a rapid development stage (with a growth rate of 3.89%), accounting for 48% of the total number of articles issued. It can be shown that the research on GHG regulation and soil improvement in cake fertilizer agriculture during 2017-2023 is getting more and more international attention. First and foremost, this is due to growing consumer interest in the production of environmentally friendly agricultural goods, of which organic farming is one of the most well-known methods for ensuring the security and caliber of agricultural output while safeguarding soil ecosystems (Jung and Choi, 2020). Furthermore, in the views of the United Nations Environment Program, Ravishkara et al., Portmann et al. and Lee et al. agricultural greenhouse gases are already indirectly affecting human health, and using environmentally friendly materials to reduce agrarian greenhouse emissions is urgent (Marcus and Nwaeze, 2024).Meanwhile, with the adoption of the Paris Agreement at the 21st United Nations Climate Change Conference, countries undertake measures to reduce carbon emissions and management (Ahmed et al., 2023b).

### 3.2 Analysis of co-operative networks

Through scientific and technological cooperation, authors can share resources, combine strengths and complement each other’s strengths in scientific research, solve major key technological problems. Therefore, this paper analyses the collaborative network of countries, authors, institutions and journals (Li et al., 2021).

#### 3.2.1 Analysis of country network cooperation

**Figure 3:**
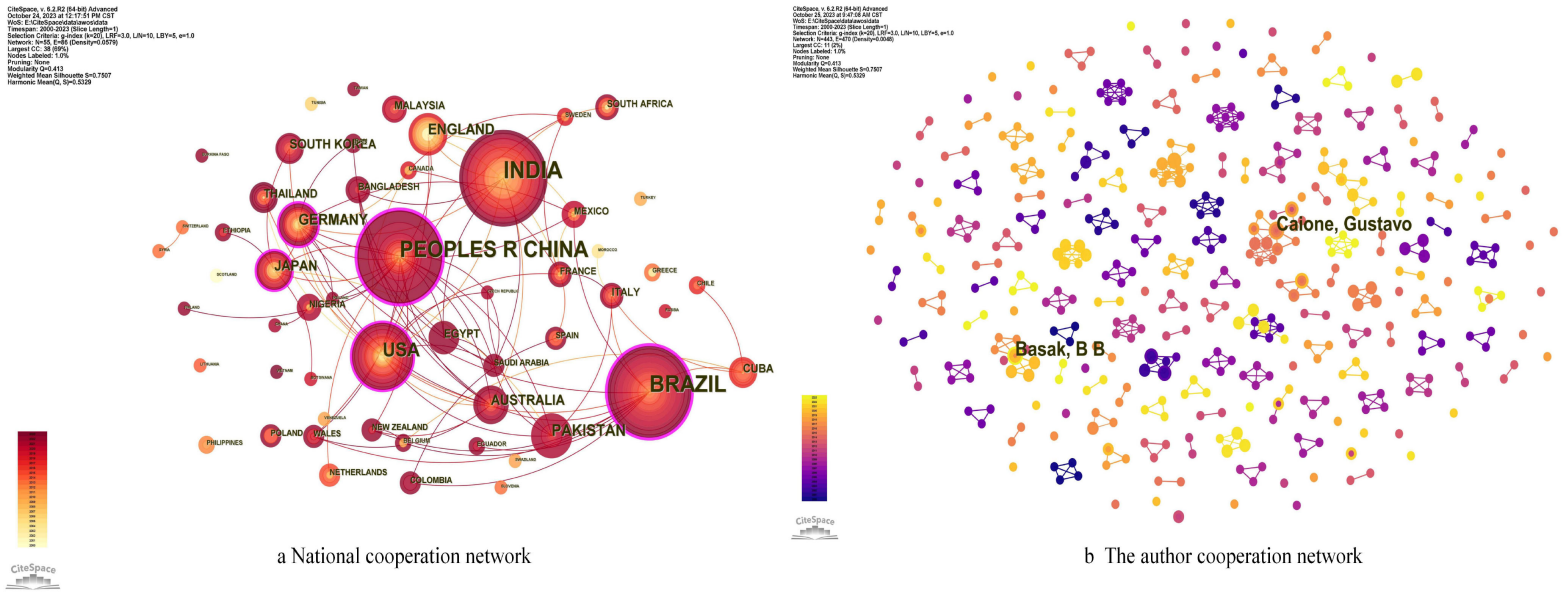
**(a, b)** National and the author cooperation network analysis.

National network cooperation analysis of the Web of Science core collection of 481 documents, set the node type for the country to analyze the literature, the national cooperation network is shown in **Figure 3a**. The nodes appearing in the figure represent different countries, the larger the radius, the greater the number of articles issued by the country, the purple outer circle indicates that the intermediary centrality is greater than 0.1. The node with a high intermediary centrality is usually connected to a different clusters, reflecting the co-operation between different countries (Freeman, 1977).

The graph yields 73 nodes with 161 lines, i.e., there are 73 countries conducting research in this field. The 3 larger nodes are the top four countries in terms of number of publications, i.e., are India with (115 publications), China with (90 publications), Brazil with (73 publications),. The top 3 countries in terms of mediational centrality are China (0.22), Brazil (0.19), and the United States (0.13).). Although China ranked second in the Web of Science core database for retrieving literature related to cake fertilizer, the intermediary centrality was located in the 1st place first, indicating that China is more active in international collaborative research, and there are different various degrees of cooperation with Brazil, the United States, and other countries, and it has a certain degree of international influence on the regulation of greenhouse gases and soil improvement in cake fertilizer. This is due to the fact that China has issued a series of relevant legal and policy documents (Bryan et al., 2018), such as the National Plan for Sustainable Agricultural Development (2015-2030), the National Green Development Plan for the 14th Five-Year Plan, and the Action Program for Peak Carbon by 2030, and that China will reach Peak Carbon by 2030 and Carbon Neutral by 2060, which have been incorporated into major national strategies.

In addition, we analyzed the primary varieties of cake fertilizer studied in the countries with more than 50 publications in the database (**Table 1**). The findings demonstrate a high degree of similarity between the primary varieties of cake fertilizers and their production processes in Brazil and India. On the other hand, China produces cake fertilizers differently from India or Brazil, both in terms of ingredients and manufacturing processes. The effect of climate is one of the primary causes of this occurrence (Baker et al., 2018). China has a largely temperate environment, whereas Brazil and India have mostly tropical climates. It is noteworthy that Brazilian researchers have given jatropha a great deal of attention because of its resistance to drought. Jatropha seed cake fertilizer (Yamada and Sentelhas, 2014), a by-product of this process, has thus been a popular study topic thus far.

**Table 1:** Status of primary cake fertilizer production in countries with >50 publications.

Country	Types of cake fertilizers	Source	Annual production volume	Production method
India	Sugarcane Filter Cake fertilizer	Sugarcane	8–10 million tons (Patil et al., 2022)	Anaerobic digestion (Patil et al., 2022)
	Castor bean cake fertilizer	The seeds of castor tree	8–10 million tons (Delvadiya et al., 2024)	Anaerobic digestion (Kalogiannis et al., 2016)
	Jatropha seed cake fertilizer	The seeds of jatropha tree	8.35million tons (Raheman and Mondal, 2012)	Anaerobic digestion (Raheman and Mondal, 2012)
	Neem seed cake fertilizer	The seeds of neem tree	1.12million tons (Abbasi et al., 2011)	Anaerobic digestion (Benelli et al., 2017)
China	Peanut cake fertilizer	Peanut	18million tons (He et al., 2021)	Fermentation (Peng et al., 2017)
	Soybean cake fertilizer	Soybean	16million tons (Zhang et al., 2024)	Fermentation (Peng et al., 2017)
	Rapeseed Cake fertilizer	Rapeseed	13 million tons (Liang et al., 2023)	Fermentation (Song et al., 2023)
	Sesame cake fertilizer	Sesame	0.60-0.65million tons (Wang et al., 2020)	Fermentation (Peng et al., 2017)
Brazil	Sugarcane Filter Cake fertilizer	sugarcane	5–9 million tons (Maranhao et al., 2024)	Anaerobic digestion (Braos et al., 2021)
	Castor bean cake fertilizer	The seeds of castor	1–1.5 million tons (dos Santos Neto et al., 2012)	Anaerobic digestion(da Silva et al., 2012)

#### 3.2.2 Author collaborative network analysis

A visual analysis of the WOS database using the Author analysis function in CiteSpace yielded the WOS Author Collaboration Network Diagram (**Figure 3b**). According to the figure, a total of 572 authors published publications from 2000 to 2024 and produced 573 connecting lines with a density of 0.0035, all of which were loosely distributed. It shows that the exploration is still in its infancy, and that the standard of research on the application of cake fertilizers to agriculture is neither uniform nor intellectually structured. The greenhouse gas control and the theory of soil improvement is not systematic enough to form a more general and consistent guiding opinion, and has certain limitations in terms of research scope, soil environment and national concern. Caione, Gustavo, and Basak, B, Hossain, Akbar, Ma, Lifeng, and Ruan, Jianyun, with the highest number of publications, with 5 publications each. Among them, Caione, Gustavo do more co-author work compared to Basak, B, while the latter only has contact with four authors. This is due to Basak, B.’s research, which focused on examining the effects of manure, castor cake fertilizer, and fungal fertilizer on Withania somnifera L. Dunal. It was shown that these nutrients could enhance the qualities of the soil and raise the quantity and caliber of Withania somnifera produced (Chaudhary et al., 2017; Basak et al., 2020), but the research methodology and content were singular and lacked an understanding of the internal mechanisms of the soil. In contrast, Caione, Gustavo focuses on the shape and transport of soil phosphorus, optimizing fertilization techniques and conducting research on maize and sugarcane. For example, Caione, Gustavo started to study the effect of rock phosphate-rich cake fertilizer on microbial populations and phosphorus content of Haplastox soils in 2014 in collaboration with González et al. (2014). In 2015 they started study the effect of cake fertilizer with different types of phosphate on the organic and inorganic phosphorus content of soils planted with sugarcane in cooperation with Caione et al. (2015), followed by a study in 2016 with González et al. (2016) to study the effect of filter cake plus microbial-enriched rock phosphate on organic and inorganic phosphorus content of soils from Haplustox and Hapludox soils on effective and adsorbed phosphorus and maize growth. In 2018 with Caione et al. (2018) studied the effect of natural phosphate, filter cake, peat and biofertilizer fertilization on non-phosphorus soil phosphorus content, foliar phosphorus content and seedling growth. Their study proved that cake fertilizers have a significant effect on the increase of g soil nutrients and crop yield. Despite of the fact that Caione Gustavo made analysis on the regulatory influences, such as cake fertilizer application methods and soil amendments, the impact on greenhouse gas regulation was neglected. Therefore, the research history of the representatives in this field was derived through the analysis of the authors’ collaborative network, which has a non-negligible role in further evaluating the research directions of the representatives.

#### 3.2.3 Analysis of the network of issuing institutions

**Figure 4:**
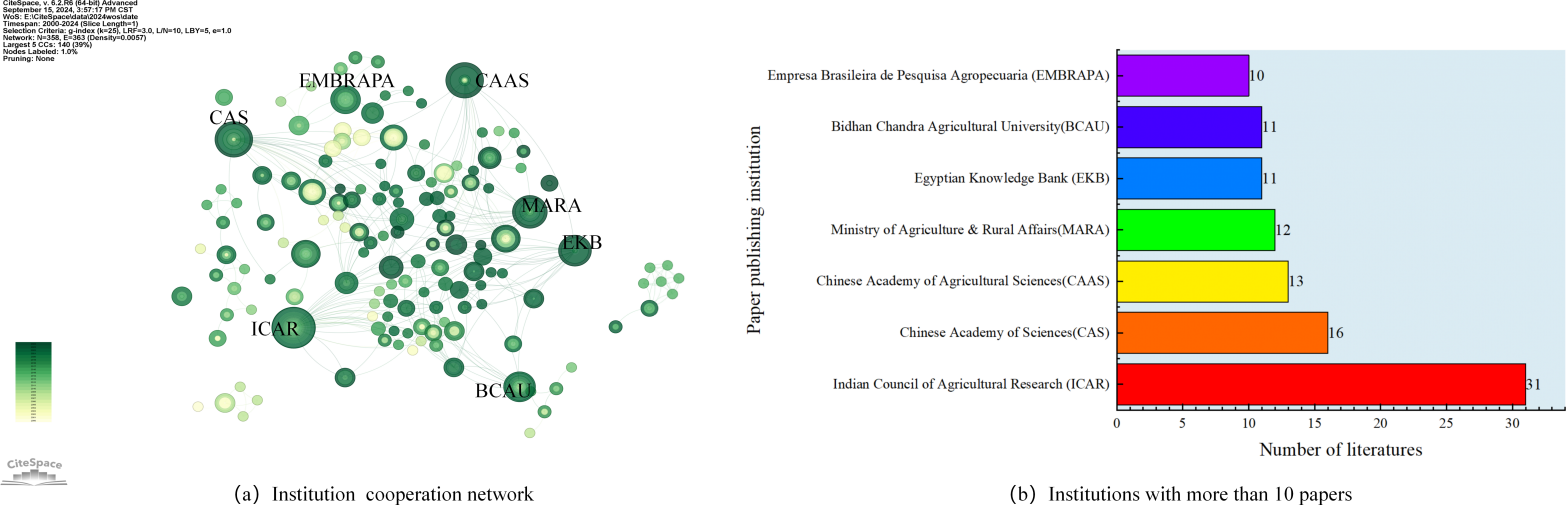
Institutional analysis diagram. **(a)** Institution cooperation network. **(b)** Institutions with more than 10 papers.

The WOS database was visualized based on the Institution analysis function in CiteSpace. This database is set to display institutions with ≥ 10 publications in the literature and the WOS Institutional Collaboration Network Diagram (**Figure 4a**). As shown in the figure, 292 institutions published their publications from 2000-2023, generating 382 connectivity lines with a density of 0.009. In the figure, University of Chinese Academy of Sciences, Empresa Brasileira de Pesquisa Agropecuaria (EMBRAPA), Chinese Academy of Agricultural Sciences, Universidade de Sao Paulo, Ministry of Agriculture & Rural Affairs, Universidade Estadual Paulista collaborates closely with other institutions. In contrast, Indian Council of Agricultural Research (ICAR) collaborates with only a small number of institutions, forming an independent but integral network, and the rest of the institutions collaborate with each other in a weak way. **Figure 4b** shows the top 7 issuing institutions in terms of the amount of literature published, of which 5 universities are listed. 5This shows that universities constitute the main research output in this field among the issuing institutions.

#### 3.2.4 Analysis of issuing journals

Analysis of the published journals is based on WOS database; the results are shown in **Table 2**. As can be seen from the table, the journals are all English journals, the impact factor is greater than 2 international journals, and the journal with the most publications is Communications in Soil Science and Plant Analysis, with a total of 16 articles published. It shows that in recent years, relevant research institutions and researchers are more inclined to publish their research in international journals.

**Table 2:** Journals with more than 6 papers.

Ranking	Journal	Number of literatures	IF
1	Communications in Soil Science and Plant Analysis	16	1.8
2	Journal of Plant Nutrition and Soil Science	13	2.5
3	Agronomy-Basel	12	3.949
	Indian journal of agricultural sciences	9	0.58
4	Soil Use and Management	8	3.8
5	International Journal of Molecular Sciences	7	5.6
6	Biology and Fertility of Soils	6	6.5

### 3.3 Keyword analysis

Keyword analysis can largely reflect the main research content, research status, research hotspot and development trend. It is mainly divided into keyword co-occurrence analysis, keyword clustering analysis, keyword emergence analysis (Lan et al., 2022).

#### 3.3.1 Keyword co-occurrence analysis

**Figure 5:**
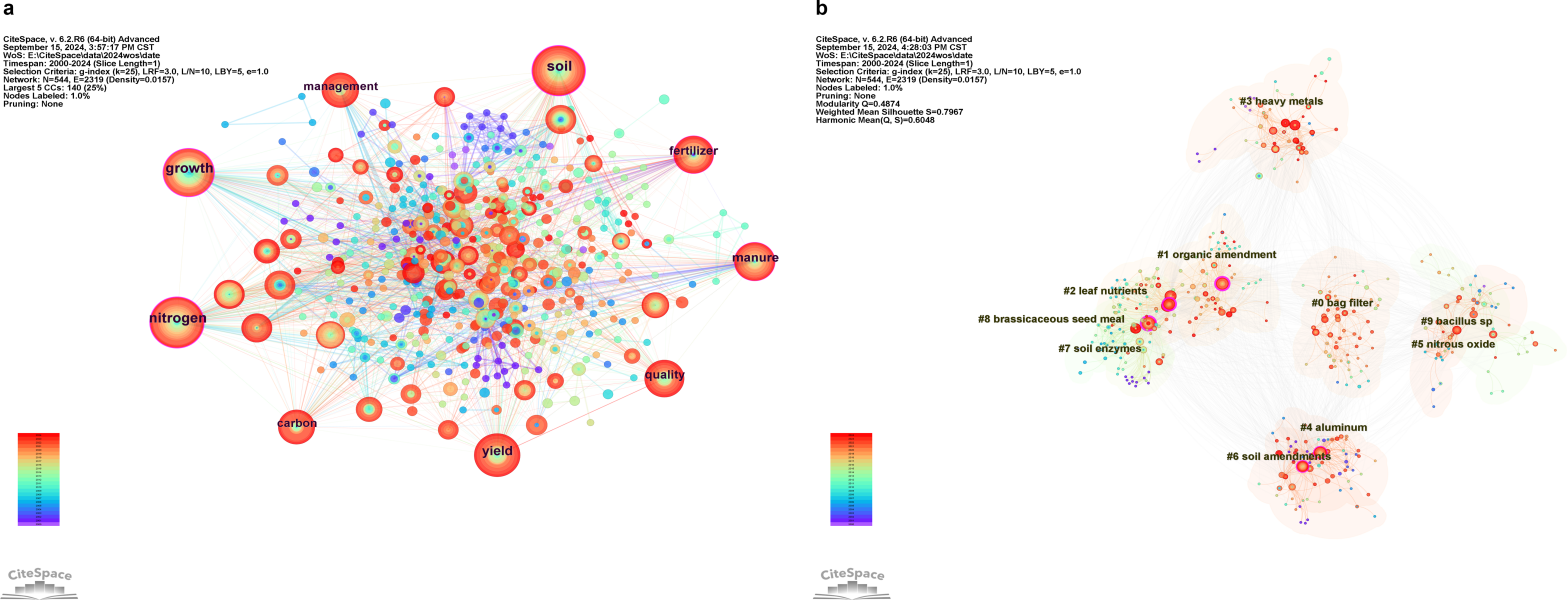
Keyword analysis. **(a)** Keyword co-occurrence graph. **(b)** Keyword clustering graph.

Keyword co-occurrence analysis aims to derive the frequency of keyword occurrences and thus determine the hot topics of research in the field. **Figure 5** exhibits the keyword co-occurrence and cluster analysis. The WOS database was analyzed to obtain a keyword co-occurrence graph using CiteSpace (**Figure 5a**). Each node in the graph represents a keyword; the larger the node, the more frequently the keyword appears; the color of the node indicates the year the keyword first appeared; the connecting line between the nodes shows the frequency with which different keywords appear in the same literature; the thicker the connecting line, the more frequently the keywords appear; the keywords displayed in the graph are those with a frequency of occurrence of ≥ 20. There are 544 nodes in the graph, i.e., 544 keywords and 2319 lines, with a density of 0.0157. According to the analysis of the statistical results, there are 8 keywords with a frequency of occurrence of more than 20, which are “nitrogen (62)”, “soil (59)”,”growth (55)”, “yield (44)”, “manure (36)”, “fertilizer (31)”, “quality (31)”, “management (28),” and “fertilizer (28)”. All these keywords have a mediational centrality greater than 0.1, from the years 2000-2024. Throughout the literature on greenhouse gas regulation and soil improvement in cake fertilizer agriculture, it can be seen that the research hotspots in the field of cake fertilizer agriculture mainly include soil fertility and crop yield and quality, and the regulation of agricultural greenhouse gases by cake fertilizer has not become a research hotspot in this field yet.

#### 3.3.2 Keyword clustering analysis

When combined with the centrality, frequency, etc. that can be acquired in various time periods of the research characteristics, keyword clustering analysis may investigate the research themes in the field of cake fertilizer, thereby intuitively portraying the hot subjects of cake fertilizer research. The WOS database was analyzed using CiteSpace, yielding the keyword clustering map (**Figure 5b**), where various color blocks correspond to distinct clustering areas. The combination of the Q and S values shows that the network clustering of the current map has rationality. The Q value is 0.49 > 0.30, indicating that the clustering analysis is scientific; the letter S value is 0.79 > 0.70, indicating that it has a high level of credibility. The clustering result graph is arranged based on the number of nodes, because the first 10 clusters contain a number greater than 20, and the 11th cluster contains a number less than 20, so **Figure 5b** only analyses the results of the first 10 clusters, i.e., 10 themes. Specific results are shown in **Table 3**. Among them, the cluster with the highest number of nodes is “ bag filiter “, indicating the theme “ bag filiter “ includes the largest number of keywords. The cluster label with the highest contour value is “ bacillus sp “, indicating that the keywords in the theme “ bacillus sp “ are more similar. This is a result of the significant responsibilities that Bacillus sp. plays in agriculture. First of all, it can be applied as a remediation agent for soil to break down soluble metals like lead, copper, zinc, nickel, and chromium (Chowdhury et al., 2024). Second, it can be applied as a biofertilizer to raise crop yields (Salehin et al., 2021) and soil quality (Ortiz and Sansinenea, 2022). Lastly, it can lower emissions of greenhouse gases (Paliwoda et al., 2023). Thus, in order to ensure the sustainable growth of agriculture and environmental preservation, fertilizer use in the future should aim to do more than only increase crop yields and soil fertility. It should also aim to lower greenhouse gas emissions.

**Table 3:** Keyword clustering results.

Cluster ID	Size	Silhouette	Mean (year)	Top Terms (LSl)
#0 bag filiter	58	0.762	2016	soil; quality; anaerobic digestate; sewage sludge; soil suppression; oil-less seed co-product
#1 organic amendment	53	0.726	2015	nitrogen mineralization; detergent-soluble organic nitrogen; greenhouse gas emissions; nutrient use efficiency
#2 leaf nutrients	52	0.722	2014	soil nutrients; organic materials; tea plantation; soil aggregate enzyme activity; chemical fertilizer
#3 heavy metals	49	0.736	2012	organic matter; microbial biomass; organic amendments; arginine ammonification; nitrogen mineralization
#4 aluminum	39	0.755	2010	sewage sludge; phosphorus; fertilizer; growth; carbon
#5 nitrous oxide	34	0.833	2008	nitrous oxide; soil fertility; mineral fertilizers; greenhouse gases; tillage
#6 soil amendment	33	0.762	2008	losses; tillage; water quality; soils; nitrogen; crop production; microbial biomass
#7 soil enzymes	32	0.843	2006	organic carbon; semiarid zones; organic wastes; triticum aestivum; irrigated soils
#8 brassicaceous seed meal	31	0.733	2010	sugarcane productivity; organic carbon; gluconacetobacter diazotrophicus; soil quality; microbial biomass
#9 bacillus sp	23	0.876	2015	fusarium oxysporum; plant growth promotion; systemic resistance; sp radicis-lycopersici

#### 3.3.3 Keyword emergence analyses

Keyword emergence examines new research trends and examines how the boundaries of a field of study change over a predetermined period of time (Xiao et al., 2023). CiteSpace was used to generate the keyword mutation table (**Table 4**), which yielded a total of six mutated terms. The keywords “growth” in 2013–2015 denote the focus of the research on crop growth. The search terms “management,” “biochar,” “matter,” and “organic fertilizer” have persisted since 2016 and suggest that research on biochar-based or biochar-containing organic fertilizers remain a hotspot.

**Table 4:** Keyword burst.

Keyword	Strength	Bursts years
Growth	3.67	2013-2015
Organic matter	3.51	2016-2020
Management	3.56	2021-2024
Biochar	4.36	2022-2024
Organic fertilizer	4.02	2022-2024
Carbon	3.88	2022-2024

### 3.4 Literature co-citation analysis

**Figure 6:**
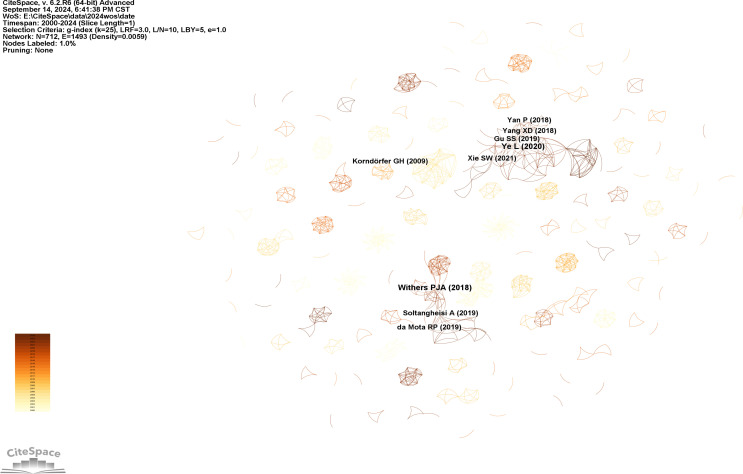
Literature co-citation network analysis diagram.

Co-citation analysis of literature can identify highly cited literature, and these highly cited articles are usually considered to be transitional between research periods (Chen, 2004). In this study, these key nodes act as “bridges” - they are bridges between papers with different research themes and co-citations with multiple papers (Yu et al., 2023). As a result, these key nodes represent hot topics in a given period of time and influence their development over time. **Figure 6** shows the literature co-citation analysis graph. The distribution characteristics of the graph are similar to the author network analysis graph, which exhibits a block distribution, and each part of the separate linkage shows that there are many topics and a wide range of research directions about the agricultural field of cake fertilizer application, which is in line with the keyword clustering results. The figure shows the literature with a co-citation frequency greater than 2, with Withers et al. (2018) and Ye et al. (2020) having the highest co-citation frequency, highlighting the importance of soil phosphorus for crop yields, as well as pointing out the importance of sustainable management of phosphorus in order to guarantee global food security (Withers et al., 2018). Through this paper, the inextricable relationship between the current theme of “soil fertility” and crop yields has been highlighted, leading to the major theme of “management”. The analysis of these key points allows speculation on the next hot topic and reflects the knowledge base of the research hot spots and frontiers.

## 4 Discussion

The above has been analyzed from keyword co-occurrences, keyword clusters, and keyword emergence to derive the research hotspots and research frontiers in different time frames, but there is no clear indication about the research content. Based on this, the above results are further discussed and some insights are provided for future research.

According to the keyword analysis, the research area of greenhouse gases and soil improvement in cake fertilizer agriculture is wide-ranging, and the main content doesn’t remain consistent. A range of influencing factors, including region, policy, science, and technology, have played a role in this transformation of agriculture over the past 20 years. The research process in the field of cake fertilizer application in agriculture over the last 20 years can be divided into three cycles, namely the pioneering period (2000-2007), the slow development period (2008-2016), and the rapid development period (2016-2023).

During the pioneering period, since excessive phosphorus can lead to degradation of water quality, the Water Quality Improvement Act in Maryland mandates P-based nutrient management for manures and biosolids by 2005 (Elliott et al., 2002). In order to regulate phosphorus in the soil, the research focused on the effects of different types of organic fertilizers applied alone or in combination with inorganic fertilizers on soil nitrogen and phosphorus transport (Withers et al., 2001; Elliott et al., 2002; Bousselhaj et al., 2004; Kaur et al., 2005) and yield quality of the crop broad bean (Abdelhamid et al., 2004). Moreover, in order to change the environmental impacts of sewage sludge, the UK pioneered the use of grassland to absorb sludge in order to reduce carbon dioxide and nitrous oxide emissions during this period. Using sludge reduces grassland greenhouse gas emissions, laying the groundwork for regulating cake fertilizers, but no mention has been made of the impact on cropland (Scott et al., 2000).

During the slow development period, The FAO’s Voluntary Guidelines for Sustainable Soil Management (Montanarella and Panagos, 2021), adopted in 2016, and China’s National Plan for Sustainable Agricultural Development (2015–2030) (Bryan et al., 2018), released in 2015, both make it clear that the development of organic agriculture should be accelerated in order to stop soil degradation and that the use of chemical fertilizers should be reduced without reducing soil fertility. Therefore, a number of studies were conducted on the effects of organic fertilizers mixed with compound fertilizers or organic fertilizers mixed with mineral fertilizers on soil microbial communities and soil improvement (Gilbert et al., 2008; Rigby et al., 2009; Dinesh et al., 2012; Murugan and Kumar, 2013; Srivastava et al., 2016), as well as the yields and quality of cash crops, including onions (Lee, 2010), sugarcane (Santos et al., 2011), and sweet oranges (Ghosh et al., 2014). A reduction in chemical fertilizer use was observed during this period, and Wang et al. conducted field experiments in southeastern China to demonstrate that organic fertilizers paired with chemical fertilizers did increase crop yields, but also significantly increased the risk of methane emissions (Wang et al., 2013). This paper examined the effects of three organic fertilizers combined with composite fertilizers on methane, nitrogen oxide, and grain yields in rice paddies. Combining chemical and organic fertilizers may increase greenhouse gas emissions for different crops, but the impact of cake fertilizer on GHG emissions has not been demonstrated and needs to be explored.

During the rapid development period, in addition to the expansion of the research content of the first two phases, research scope is widened, as well as the increasing limitations of traditional materials. The development of the new energy and new material industries, as well as the increased demand for new materials and energy, has led to the emergence of new materials into agricultural research of cake fertilizer application. Meanwhile, the Chinese government has issued a series of legal and policy documents to promote the replacement of chemical fertilizers with organic fertilizers (Lin B. et al., 2022). For example, the Action Plan for Replacing Chemical Fertilizers by Organic Fertilizers in Fruits, Vegetables, and Tea Plantations and the Opinions on Innovating System and Mechanism to Promote Agricultural Green Development, both released in 2017. And in December 2019, the European Commission published the European Green Deal (EGD) (Dupont and Torney, 2021). Research demonstrating that using environmentally friendly products along with agricultural management increases productivity while also improving crop yields and soil fertility. For example, Zhang et al. studied agronomic measures to improve rice yield and nitrogen use efficiency by adding rapeseed cake fertilizer, increasing the frequency of fertilizer application and increasing the frequency of irrigation (Zhang et al., 2017); Mortierella elongata, a dominant fungal strain, was studied for its phosphorus uptake, its response to organic fertilizer, and its role in growth promotion by (Li et al. (2018); Zhang et al. studied the optimization of integrated cultivation management to improve crop yield and nitrogen use efficiency by exploring different planting densities, irrigation levels, rapeseed cake fertilizer amounts and other management practices (Zhang et al., 2019); Manzoor et al. addressed the effect of biochar, rapeseed cake meal and chemical fertilizer formulation on root growth, nutrient utilization and yield of tea tree (Manzoor et al., 2022). Therefore, more in-depth investigation of the above new materials and agronomic practices is needed to explore the potential of the effects on crop quality and soil fertility, and to provide more possibilities for soil improvement in cake manure agriculture.

Despite rapid development, research on the impact of cake fertilizers on greenhouse gas regulation in agriculture has continued. For example, A study conducted by Zhou et al. examined the effects of rapeseed cake fertilizer and green manure fertilizer on citrus orchard yields and nitrogen oxide emissions. Based on these findings, citrus orchards can achieve sustainable productivity and protect their environment if they apply rapeseed cake fertilizer and reduce fertilizer use by 30% (Zhou et al., 2022). Grutzmacher et al. demonstrated the potential of biochar to reduce fertilizer-induced nitrous oxide emissions by exploring the effects of biochar with chicken manure, sewage sludge, press tree chips and filter cake on nitrous oxide emissions, with sludge cake fertilizer with biochar reducing soil fertilizer emissions by 87% (Grutzmacher et al., 2018). Furthermore, the Life Cycle Assessment (LCA) evaluation technique has demonstrated that biochar significantly reduces greenhouse gas emissions (Osman et al., 2024). This is owing to the fact that biochar may stabilize carbon in soil used in agriculture (Osman et al., 2022). However, there is a lack of using LCA on cake fertilizer to assess the impact of cake fertilizer on environmental factors, as well as resource consumption and environmental emissions during the production and use of cake fertilizer. Therefore, it’s critical to improve research on GHG emissions and consumption during the manufacture and application of cake fertilizer in addition to investigating the effects of mixing cake fertilizer with environmentally friendly products on GHGs.

Based on the above content research, cake fertilizer should be applied in a way that maximizes its benefits for crop growth and soil management. First, Cake fertilizers should be used in conjunction with biochar to meet environmental criteria, followed by the application of chemical fertilizers to maximize nutrient uptake and reduce the danger of nutrient imbalances, according on local soil and crop needs. Second, in terms of fertilizer amount and timing, the initial application is made one week before the planting of the crop. Depending on the soil type, crop type and management type in different areas, the amount of fertilizer applied varies. It is recommended to calculate the appropriate ratio of nitrogen, phosphorus and potassium based on the nutrients in the cake fertilizer. In circumstances where crop fertilizer demand is high and previous soil nutrient loss is excessive, a second application might be applied to ensure crop yield and quality and should be carefully managed to avoid potential environmental pollution. It is suggested to conduct soil tests before applying cake fertilizer to determine the appropriate application rate. Finally, adopting precision agriculture tools (e.g., IoT-based soil sensors, drone mapping) can optimize cake fertilizer application by dynamically adjusting doses based on real-time soil nutrient status and crop phenology.

Moreover, numerous experiments have shown that cake fertilizers significantly improve soil and reduce greenhouse gas emissions. However, there is a lack of potential mechanisms caused by chemical reactions or physical processes. Advances in chemistry and allied subjects, as well as a thorough understanding of molecular systems, can result from the combination of experimental data and computational chemistry approaches, according to certain research (Ahmed et al., 2020). For instance, the intricate impacts of pH on phosphorus binding and transport in soil were demonstrated, by Ahmed et al.’s molecular modeling and simulation studies (Ahmed et al., 2023a). Shaheen et al. revealed the complex effects of metal oxides on phosphorus morphology and transport in soil using soil spectroscopic and molecular methods(quantum chemical calculations (QCC)), providing more detailed theoretical principles for phosphorus management in soil (Shaheen et al., 2022). In addition, there are studies on the use of predictive modeling in machine learning to assess soil fertility and productivity (Helfer et al., 2020), as well as combining computational chemistry and machine learning to explore the effects of variables on biochar (Osman et al., 2023). Nevertheless, there isn’t much research on machine learning and computational chemistry in relation to cake fertilizer. In order to investigate the chemical reactions and physical processes of cake fertilizers on soils, it is crucial that future study on the topic combine cake fertilizers with adjacent fields like computational chemistry. Additionally, machine learning models should be utilized to assess and forecast experimental data.

Bibliometrics was used to systematically summarize the study. WOS database, which contains articles, citations, etc., was used for literature bibliometrics. As a result, the results of this study may vary based on the type of database, the keywords used, and the time of the search. In order to assure the accuracy of the data and minimize any bias, this article offers a thorough reading and screening of the chosen data in addition to a precise account of the search method and duration of the study. Furthermore, in order to present a complete picture, this work integrates expert opinion in addition to using bibliometrics for research. This study aims to impartially and objectively analyze the field’s developmental dynamics over a certain time period using these metrics.

## 5 Conclusions and recommendations

The research on greenhouse gases and soil improvement in cake fertilizer agriculture during 2000-2023 is intensifying, with India ranking first in terms of the number of published articles. Having a certain degree of international influence on greenhouse gases and soil, China is more active in international collaborative research. The most influential journal is Journal of Plant Nutrition and Soil Science, Caione, Gustavo and Basak, B B as core authors. The development trend is as follows. In the pioneering period, organic fertilizers alone or in combination with inorganic fertilizers are being studied for their effects on soil nitrogen and phosphorus transport, as well as soil fertility. In the slow development period, the main focus is on the effects of organic fertilizers and mineral fertilizers on soil microbes and soil improvement. In the rapid development period, the scope of research expands; in addition to the research content of the previous two phases, there are new materials, as well as setting different water, soil conditions and other agronomic conditions.

We make the following recommendations about the development of cake fertilizers for soil improvement and greenhouse gas reduction based on the information and debates in the article.

1. To investigate the effects of other chemicals in addition to organic fertilizers or even integrated agronomic approaches on soil improvement, in addition to increasing the influence on the production and quality of other crops.
2. To expand the body of knowledge on the greenhouse gas effects of cake fertilizers both during manufacture and during usage, as well as to look into the effects of cake fertilizers when combined with eco-friendly ingredients.
3. To establish connections between cake fertilizers and adjacent fields like computational chemistry in order to look at the chemical and physical processes that cake fertilizers engage in on soils, as well as to use machine learning models to interpret and forecast experiment results.
4. To analyze the effects of cake fertilizers on soil improvement and greenhouse gas emissions using a range of research techniques (meta-analysis and LCA) in order to give a thorough assessment of cake fertilizer using these two metrics.

## Data Availability

The original contributions presented in the study are included in the article/supplementary material. Further inquiries can be directed to the corresponding author.
